# Angioid Streaks Remain a Challenge in Diagnosis, Management, and Treatment

**DOI:** 10.3390/vision8010010

**Published:** 2024-03-05

**Authors:** Georgios Tsokolas, Charalambos Tossounis, Straton Tyradellis, Lorenzo Motta, Georgios D. Panos, Theo Empeslidis

**Affiliations:** 1 Ophthalmology Department, Royal Bournemouth Hospital, University Hospitals Dorset NHS Foundation Trust, Castle Lane East, Bournemouth BH7 7DW, UK; charalambos.tossounis@uhd.nhs.uk; 2 Ophthalmology Department, Leicester Royal Infirmary, University Hospitals Leicester NHS Trust, Infirmary Square, Leicester LE1 5WW, UK; s.tyradellis@nhs.net; 3 Department of Ophthalmology, School of Medicine, University of Padova, 35121 Padova, Italy; lorenzomotta@hotmail.com; 4 Department of Ophthalmology, Queen’s Medical Centre, Nottingham University Hospitals, Nottingham NG7 2UH, UK; 5 Division of Ophthalmology and Visual Sciences, School of Medicine, University of Nottingham, Nottingham NG7 2UH, UK; 6 Stoneygate Eye Hospital, Leicester LE1 2EG, UK; theo@eshcs.co.uk

**Keywords:** angioid streaks, pseudoxanthoma elasticum, aflibercept, bevacizumab, Bruch’s membrane, brolucizumab, faricimab, hemoglobinopathies, Paget’s disease, *peau d’orange*, ranibizumab

## Abstract

**Aim**: Angioid streaks (ASs) are a rare retinal condition and compromise visual acuity when complicated with choroidal neovascularization (CNV). They represent crack-like dehiscences at the level of the Bruch’s membrane. This objective narrative review aims to provide an overview of pathophysiology, current treatment modalities, and future perspectives on this condition. **Materials and Methods**: A literature search was performed using “PubMed”, “Web of Science”, “Scopus”, “ScienceDirect”, “Google Scholar”, “medRxiv”, and “bioRxiv.” **Results**: ASs may be idiopathic, but they are also associated with systemic conditions, such as pseudoxanthoma elasticum, hereditary hemoglobinopathies, or Paget’s disease. Currently, the main treatment is the use of anti-vascular endothelial growth factors (anti-VEGF) to treat secondary CNV, which is the major complication observed in this condition. If CNV is detected and treated promptly, patients with ASs have a good chance of maintaining functional vision. Other treatment modalities have been tried but have shown limited benefit and, therefore, have not managed to be more widely accepted. **Conclusion:** In summary, although there is no definitive cure yet, the use of anti-VEGF treatment for secondary CNV has provided the opportunity to maintain functional vision in individuals with AS, provided that CNV is detected and treated early.

## 1. Introduction

Angioid streaks (ASs) are a very rare condition. They represent crack-like lesions at the level of the Bruch’s membrane [1]. This descriptive review aims to provide a concise overview of pathophysiology, current treatment modalities, and future perspectives.

## 2. Materials and Methods

A literature search was performed using “PubMed”, “Web of Science”, “Scopus”, “ScienceDirect”, “Google Scholar”, “medRxiv”, and “bioRxiv.”. The main keywords for the literature search were “Angioid Streaks (AS)”, “Choroidal Neovascular Membrane (CNV)”, “Haemoglobinopathies”, “Hereditary Collagen Disorders”, “Photodynamic Therapy (PDT)”, “Pxeudoxanthoma Elasticum (PXE), “Vascular Endothelial Growth Factor (VEGF)”, “Bevacizumab”, “Brolucizumab”, “Ranibizumab”, and “Faricimab”. The authors attempted to collect data from manuscripts that were mainly published in the past 15 years up to the present day so that the most recent and up-to-date information on ASs could be incorporated into this manuscript. Nevertheless, published manuscripts from more than 15 years ago were also used, as they contained pertinent information about AS. No language restrictions were applied. As this is a narrative review, no ethical approval was required.

## 3. Narrative Review Findings 

### 3.1. Historical Background

The first description of ASs in the literature was published by Robert Doyne in 1889 [2,3]. Doyne examined the fundus of a patient with retinal hemorrhages due to trauma and reported “irregular peri-papillary jagged lines, extending from the disk margin to the retinal periphery” [2,3]. In 1891, the German ophthalmologist Otto Plange, while being an ophthalmology resident at Bochum and independently from Doyne, reported a patient who developed “intra-retinal pigmented deposits following hemorrhage” [4,5]. A year later, in 1892, Knapp coined the term “angioid” due to the popular belief at the time suggesting that these lines were of vascular origin [3,6]. 

However, in 1917, Alfons Kofler was the first ophthalmologist to correctly propose that ASs are attributed to breaks in the Bruch membrane [3,5,7,8]. This postulation was further supported by histological studies in later years [5,9,10,11,12,13,14]. Furthermore, in 2009, Charbel Issa and colleagues confirmed Kofler’s hypothesis using in vivo imaging techniques [5,15]. In 1941, Scholz published the first thorough review of this condition based on the available literature at the time [5,16]. 

Regarding the fundus examination findings in patients with ASs, the term *peau d’orange*, which is a very common clinical fundus finding in AS patients, was first proposed by Smith and colleagues in 1964 [5,17]. In 2003, Gass introduced the term “comet tail lesions” for the characteristic spot-like chorioretinal lesions with a tail pointing toward the posterior pole [5,18].

The first hypothesis of a potential association of this ocular pathological entity with a systemic disease was suggested by Hallopeau and Laffitte in 1903 [5,19]. The confirmation of the syndromic association between the ocular and systemic clinical phenotypes came 16 years later, in 1929, thanks to the collaboration of Swedish ophthalmologist Ester Grönblad and dermatologist James Strandberg [5,20,21]. This collaboration confirmed the association of ASs with the condition pseudoxanthoma elasticum (PXE), and this is why this condition is also known as Grönblad–Strandberg syndrome. PXE, which is the most common association among ASs [3], will be discussed later in this manuscript.

### 3.2. Prevalence

The reported prevalence/frequency of ASs vary depending on the systemic association and studied age group [3,8]. Chatziralli et al. suggested that the prevalence of ASs in PXE patients varies between 59% and 87% [3]. Nevertheless, Charbel Issa suggested that these numbers may not reflect the true prevalence of PXE [8]. Charbel Issa suggested that the differences in the percentages can be attributed to the age of the investigated patients, the experience of the examiners, and the imaging techniques used to diagnose ASs [8]. He further commented that the frequency of ASs should not be based on the diagnostic methods used to diagnose PXE (e.g., skin biopsy) [8]. Furthermore, he suggested that the frequency of any underlying associated systemic disease will depend on the clinician’s effort and use of different tests to identify such disease, which can vary from skin biopsy to genetic testing [8]. However, in a more recent study from the United States published in June 2023 that represents the largest collection of AS patients to date [22], Nadelmann et al. reported a percentage of 12.3% [22].

In Paget’s disease, Dabbs et al. reported that the frequency of ASs may vary from 8% to 15% [3,23]. On the other hand, Nadelmann et al. reported a much smaller percentage of 0.3% [22].

In sickle cell hemoglobinopathies, the prevalence of ASs fluctuates between 0.9% and 6%, depending on the sample size of the study [3,24,25,26,27,28]. Finally, the frequency of ASs in beta thalassemia increases with age and may reach up to 30% in patients with the disease above the age of 50 [8,29]. On the other hand, Nadelmann et al. reported that the prevalence rate of hemoglobinopathies was 1.6% [22].

### 3.3. Systemic Associations

Over 50% of AS patients seem to have an underlying systemic condition [3]. PXE is the most common systemic condition associated with ASs [3]. It affects not only the skin and eyes but also the gastrointestinal tract and the cardiovascular system, leading to life-threatening complications [3]. Paget disease and hereditary hemoglobinopathies have also been associated with ASs [3].

It has been suggested previously that there is also an association of ASs with hereditary collagen disorders, including Ehlers–Danlos (ED) and Marfan. However, the association between ASs and ED was made after a single publication in 1966, where the authors reported two members of the same family having the condition [8,30]. Nevertheless, more recent studies suggest that ED patients do not usually exhibit ASs [8,31]. The same applies for Marfan syndrome and numerous systemic diseases, as reported in other manuscripts [3,8,32,33]. Charbel Issa suggests that the presence of ASs may be purely coincidental in these conditions [8]. He also suggested that a larger number of cohorts of patients with prolonged and consistent follow-up are required to uncover a true link between ASs and other systemic diseases [8]. Despite emerging literature suggesting a weak link between Ehler–Danlos syndrome and AS, the mnemonic PEPSI, representing pseudoxanthoma elasticum, Ehler–Danlos syndrome, Paget’s disease of bone, sickle cell disease, and other hemoglobinopathies, and idiopathic cases, remains a valuable tool for recalling the spectrum of conditions associated with angioid streaks [31].

Nadelmann et al. suggested that the classical associations of ASs with PXE, hemoglobinopathies, and Paget were observed at a far lower rate than in previous studies [22]. Moreover, they suggested that there was a statistically higher prevalence of the following less classically associated diseases among patients with ASs compared to controls: hereditary spherocytosis (1.7% vs. 0.6%, *p* < 0.001), connective tissue disease (1.0% vs. 0.3%, *p* < 0.001), and non-exudative age-related macular degeneration (AMD) (33.9% vs. 10.6%, *p* < 0.001) [22]. This suggests potential new connections between ASs and systemic diseases, potentially illuminating the mechanisms behind AS formation. Concurrent with these findings, ASs are also noted for the abnormal activation and dysregulation of mineralization processes in the elastin-rich Bruch’s membrane (BrM), leading to its thickening and increased brittleness. This process is accompanied by the activation and migration of the retinal pigment epithelium (RPE) to different retinal layers, which, upon disruption of BrM’s mechanical integrity, results in the degeneration and atrophy of the overlying RPE. These insights may offer further understanding of the underlying pathways leading to the development of ASs [3,22].

In summary, the available data regarding the prevalence of ASs and their connection with systemic disorders have discrepancies, which may be attributed to the differences in age groups, duration of follow-up, number of patients studied, and ethnic diversity of the studied population. This further reinforces the suggestion made by Charbel Issa regarding larger cohorts of patients with prolonged and consistent follow-up while attempting to establish a true association between ASs and other systemic disorders [8].

### 3.4. Genetics

ASs do not represent a hereditary condition per se [8]. The mode of inheritance is known for most of the systemic underlying disorders associated with ASs (PXE and hemoglobinopathies included) [8]. The bulk of the available data regarding the genetics of ASs originates from studies that focused on PXE patients [3].

PXE is an autosomal recessive condition caused by mutations in the *ABCC6* gene [5,34,35]. This gene is predominantly expressed in hepatocytes and kidney cells and, to a lesser degree, in the tissues linked to the PXE clinical phenotype [5,36]. It encodes a protein that serves as a trans-membrane transporter of anionic small molecular weight conjugates [5,32,37,38,39]. In PXE, the various clinical manifestations are attributed to the increased amount of calcification of connective tissue rich in elastic fibers [5]. Based on all the above details, it has been postulated that the trans-membrane transporter of anionic small molecular weight conjugates encoded by the *ABCC6* gene may serve as a shuttle for one or more molecules that inhibit the calcification of elastic fibers [5]. This hypothesis has been further supported by data originating from a mouse animal model [5,40,41]. Numerous mutations have been described to cause dysfunction in the trans-membrane transporter, leading to the clinical manifestation of the PXE phenotype [5,35,37,42,43,44,45].

Finally, there are isolated cases of ASs where there is no obvious link to an underlying systemic disorder. These are known as idiopathic [8]. Idiopathic ASs are rare and usually occur in older patients [8]. It has been suggested that they may represent a “form fruste” due to *ABCC6* haploinsufficiency [8,46] and may have multifactorial origin [8,46]. Alternatively, they may represent a separate systemic or ocular clinical entity, which is still unknown [8].

### 3.5. Pathophysiology of the Condition and Histological Findings

Bruch’s membrane is located between the retinal pigment epithelium (RPE) layer and the choriocapillaris and allows the transportation of various nutrients and metabolites between these two layers [3]. It also serves as an important barrier between the choroidal circulation and the outer retinal layers [3,47]. To serve these two functions, Bruch’s membrane contains a remarkable amount of elastin and collagen fibers, which provide its unique biomechanical properties [3].

As mentioned above, the ABCC6 gene encodes a protein that serves as a trans-membrane transporter of anionic small molecular weight conjugates [5,32,37,38,39]. It has been postulated that the trans-membrane transporter of anionic small molecular weight conjugates encoded by the ABCC6 gene may serve as a shuttle for one or more molecules that inhibit the calcification of elastic fibers [5]. This hypothesis has been further supported by data originating from a mouse animal model [5,40,41]. The various mutations in the ABCC6 gene induce disruption of this metabolic pathway [3,5]. As a result, numerous molecules of calcium minerals accumulate at the level of Bruch’s membrane [3,5]. A calcified Bruch’s membrane loses its elastic properties and becomes more brittle, leading to the appearance of the crack-like lesions that we know as ASs [3,5,48]. It is quite interesting that the phenotypic appearance of ASs is similar irrespective of the underlying systemic disorder, which further implies a strong link between ASs and the metabolic pathway described above [3,49]. The localized disruption of Bruch’s membrane integrity compromises the retina–RPE barrier, allowing communication of the choriocapillaris with the outer retina layers, which can trigger CNV formation [3]. CNV is the major complication of Ass, leading to a disciform scar and subsequent loss of vision [3]. Finally, it has also been postulated that peripapillary atrophy or peripapillary choroidal sclerosis may also be predisposing factors for the manifestation of ASs [3,50].

Regarding the histopathology of ASs, the first data came in 1938 from Böck, who confirmed Kofler’s hypothesis [3,5,7,9]. Böck studied specimens from two patients with PXE [3,5,7,9]. He observed the crack-type lesions, which were more prominent around the peripapillary region and not around the ora serrata [3,9]. He also commented on the loss of elasticity of the choriocapillaris and ciliary vascular network [3,9]. In later years, more histopathology reports and articles were released, supporting Böck’s observations even further [5,9,10,11,12,13,14].

### 3.6. Signs and Symptoms

One of the most striking clinical features of ASs is the *peau d’orange* mottled appearance of the fundus [3,5]. This important clinical finding seems to be pathognomonic for PXE [3,51]. It represents the earliest fundus finding in PXE individuals prior to the development of ASs [5]. At the initial stages of the disease, *peau d’orange* is confined to the posterior pole, but it may be seen more peripherally at later stages of the disease [5]. According to Gliem et al., at the later stages of the disease, the initially observed whitish or opaque fundus reflex may become more uniform posteriorly to the area of *peau d’orange* [5]. Peripheral to the *peau d’orange* region, the fundus reflex often becomes darker [5]. These changes in the fundus reflex between the posterior pole and the peripheral retina are quite prominent in more darkly pigmented patients, whereas in lightly pigmented individuals, they may go unnoticed even in the very late stages of the disease [5,51]. Moreover, it has been suggested that the areas of *peau d’orange* may represent a transitional region of interrupted calcification of the Bruch’s membrane [5,52]. When the fundus reflex obtains a more uniform pattern, this might suggest that the calcification becomes continuous [5,52]. *Peau d’orange* appears to spread in a circumferential, though not symmetrical, pattern [5,52] and seems to be more evident in the temporal mid-peripheral retina [5,52]. Finally, it has been proposed that it increases with age, but currently there is not enough data to support this theory further [5,52].

ASs may be of a red-brown, gray, pigmented, or mixed color [3]. The color is independent of the associated underlying systemic condition and any additional fundus changes [3,53]. Their presence can be observed entirely around the optic disc, or they can extend more peripherally or within the posterior pole [3]. If they extend within the posterior pole, then they are more likely to encroach into the fovea, and if this occurs, then the patients are very likely to complain about reduced vision, central scotoma, or distortion [3,32,49]. If the fovea is not involved, the patient may remain asymptomatic, and in such cases, the presence of ASs may be discovered incidentally [3]. 

Recurrent intra-retinal or sub-retinal hemorrhages are a common clinical finding [3]. They usually occur along the area of the streaks and are seen very frequently within the posterior pole, leading to a reduction in vision [3,54]. In addition, due to the fragility of Bruch’s membrane, choroidal rupture may occur either spontaneously or due to an ocular trauma [3,49]. Therefore, AS patients should be encouraged to avoid strenuous physical activities (for example, contact sports) and to wear protective goggles [5].

CNV formation within the macular region is the most frequent complication and is the most important cause of reduced vision, metamorphopsia, or blindness in AS patients [3,5]. Chatziralli et al. reported a frequency varying from 42% to 86% [3]. However, the percentage may fluctuate depending on the studied cohort of patients, their age, and their underlying condition [8,29,55]. Unlike hemoglobinopathies, CNV formation is quite a prominent clinical feature in PXE [3,56].

CNV can be either classic (located between the RPE and photoreceptor layer) or occult (underneath the RPE layer) [5,57]. The high incidence of CNV within the macular region can be explained by the increased frequency of AS occurrence within the posterior pole [5]. CNV can be accompanied by the presence of retinal hemorrhages, exudation, edema, retinal epithelium detachment, subretinal fibrosis, and chorioretinal atrophy [3,5]. Nakagawa et al. have suggested that the classic CNV can be more detrimental to visual function compared to the occult [5,57]. Therefore, prompt recognition of CNV formation is crucial for the initiation of treatment as an attempt to preserve vision in such patients [3,5,57]. Intravitreal injections with anti-VEGF agents are the cornerstone of treatment, and they will be discussed later in this article. Rarely, eccentric CNV may develop with minimal or no impact on the overall visual function [5]. If a CNV affecting the macular region is left untreated or not recognized in a timely manner, a macular scar will ensue, resulting in irreversible visual loss [3,56]. 

Another important issue regarding CNV formation secondary to ASs is the frequency with which recurrence occurs [3]. CNV may reactivate in the same location years after treatment [3]. In addition, new, fresh CNV may appear from different areas of the retina located away from the original CNV [3]. The visual outcome will largely depend on the location of CNV, the age of the patient during the original onset of symptoms, and the concomitant presence of an underlying systemic condition [3]. Finally, it has been observed that the decline in visual function in CNV secondary to ASs occurs at a faster rate than in CNV due to wet AMD [3,49].

In more recent years, the development of polypoidal choroidal vasculopathy (PCV) secondary to ASs has been reported [3,5,57,58,59,60]. They seem to have developed in patients with ASs associated with PXE [3,5,57,58,59,60]. Furthermore, the coexistence of retinal telangiectasia with ASs associated with sickle cell hemoglobinopathy has been reported [3,61].

Another fundus examination finding that has been reported to be pathognomonic for AS associated with PXE is the presence of comet lesions with or without tails [5,18]. According to Gliem et al., these are white, solitary nodular sub-retinal lesions with a tapering white tail extending posteriorly of the comet body and pointing toward the optic disk [5]. The body of these lesions may also contain a variable amount of pigmentation at the edges [5]. Sometimes, a cluster of such lesions may be observed in the fundus of PXE individuals [5]. They are usually located at the mid-peripheral retina, and they are the only PXE-associated retinal finding that occurs more peripherally than *peau d’orange* [5,18,52]. Comet lesions with or without tails have also been described in heterozygous carriers of ABCC6 mutations [5,62]. This observation might be of great importance, especially in young individuals that are yet to develop ASs [5].

Moreover, PXE patients may exhibit pattern dystrophy-type changes along with the presence of ASs [5]. The frequency of such changes has been reported to vary between 10% and 70% [5,63,64]. According to a classification system suggested by Agarwal et al., these changes may be divided into vitelliform, butterfly, and reticular dystrophy, or fundus flavimaculatus or pulverulentus [5,63,64,65,66,67]. Some studies have suggested that the presence of these pattern dystrophy type changes might be a valuable and reliable prognostic factor regarding secondary CNV manifestation, but at the same time acknowledge that more longitudinal data are needed to further corroborate this hypothesis [5,63].

Finally, optic disc drusen may also be observed in the fundus examination of patients with Ass associated with underlying PXE [5]. The prevalence of optic disc drusen in PXE fluctuates from 6 to 8% [5,63,68] to just over 20% [5,69], whereas in the general population the prevalence is estimated to be approximately 0.3% [5]. Although it is not entirely clear why this occurs, it has been postulated that the abnormal mineralization pathway-triggered ABCC6 mutations may be implicated in the formation of optic disc drusen in PXE individuals [5]. It is recommended to document the presence of drusen and observe their evolution over time using various imaging modalities, namely ultrasonography and autofluorescence [5,62,63].

### 3.7. Imaging of Angioid Streaks

Advancements in the various imaging techniques over the past few years have allowed for a more detailed depiction of ASs. These imaging modalities are very useful in the diagnosis and monitoring of patients with ASs [3]. Each one of them will be described in greater detail below. The data presented in this section of our manuscript originate mainly from published articles related to PXE patients, since PXE is the archetype disease for ASs and is the most common systemic disorder associated with ASs.

**(I) CONFOCAL REFLECTANCE IMAGING.**

**Figure 1:**
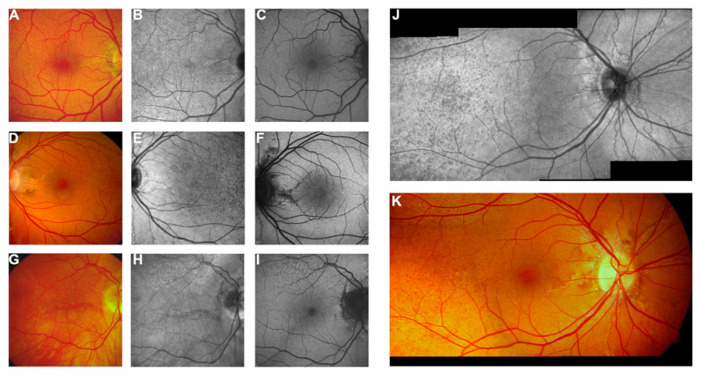
Near-infrared reflectance (NIR) imaging and 488 nm fundus autofluorescence in pseudoxanthoma elasticum. Angioid streaks and *peau d’orange* are best and most reliably visible on NIR reflectance imaging (**B**,**E**,**J**), correlating well with findings on funduscopy (**A**,**D**,**G**,**K**). Peau d’orange is usually not discernible on 488 nm fundus autofluorescence images (**C**,**F**). Angioid streaks may present with reduced autofluorescence (**C**,**F**) but may as well remain undetected on autofluorescence imaging (**H**,**I**). Note the reticular drusen on NIR reflectance and 488 nm autofluorescence, which are sometimes associated with pseudoxanthoma elasticum (**H**,**I**) [5].

Near-infrared reflectance (NIR) (also known as confocal reflectance imaging) is a non-invasive technique that can reveal the presence of ASs even if they go unnoticed on colored imaging [3,5,15,62]. Infrared imaging uses a light wavelength of 790 nm [5]. This wavelength exhibits slow absorption by the melanin within the RPE cells [5]. Along with the capabilities of the confocal imaging apparatuses, this imaging technique allows for more detailed imaging of any changes hiding underneath the RPE layer [5]. With NIR imaging, ASs appear as irregular jagged lines or fissures around the optic nerve head [5,15,62] (Figure 1). In addition, NIR is very useful in delineating the extension of the *peau d’orange* [5,15,62] (Figure 1). In fact, near-infrared imaging has shown that *peau d’orange* extends beyond the visible area of colored imaging [5,15,62], and in the absence of significant macular atrophy or scarring, it may reach up to the mid-peripheral retina or the equator [5,15,62]. Finally, undetected comet lesions on colored imaging may be unveiled with the use of NIR [5].

**(II) FUNDUS AUTOFLUORESCENCE (FAF).**

**Figure 2:**
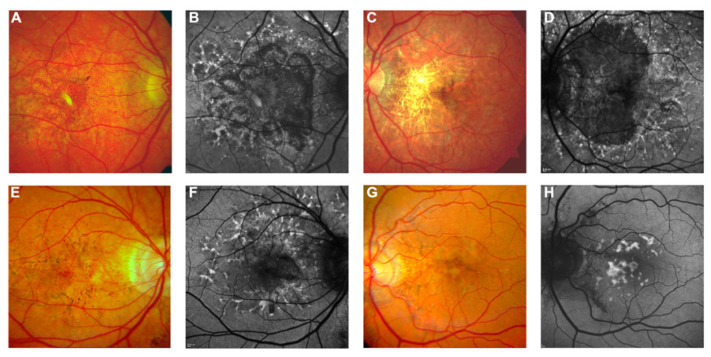
Pattern dystrophy-like changes and atrophy in pseudoxanthoma elasticum on 488 nm fundus autofluorescence imaging. Atrophic lesions and pattern dystrophy-like changes are typical features of advanced pseudoxanthoma elasticum. Compared to funduscopic images (**A**,**C**,**E**,**G**), these lesions are best visible on 488 nm fundus autofluorescence images (**B**,**D**,**F**,**H**). Pattern dystrophy-like lesions encompass different patterns of increased autofluorescence. Depending on the stage of pattern dystrophy changes, atrophy of the retinal pigment epithelium with reduced autofluorescence may be present (**B**,**D**,**F**) [5].

Fundus autofluorescence (FAF) is also a non-invasive imaging modality that uses a blue or green excitation light that allows the evaluation of the function of the RPE layer in vivo [5]. FAF detects light that is emitted from the lipofuscin within the RPE cells, reflecting their metabolic activity and health [3,66,67,70,71]. ASs may exhibit both hyper- and hypo-autofluorescence [5]. Hypo-autofluorescence suggests the loss of the RPE layer and more severe tissue damage [5]. Finger et al. and De Zaeytijd et al. described the “parastreak phenomenon”, which consists of the presence of focal spots of hyper-autofluorescence at the later edges of the streaks adopting a wing pattern [5,62,63]. They seem to represent actual spots of hyperpigmentation that are visible on colored imaging [5,62,63]. FAF may also demonstrate changes compatible with pattern dystrophy, as mentioned previously in the text [5,63,64,65,66,67] (Figure 2). 

Comet lesions exhibit hyper-autofluorescence [5]. It is yet to be clarified whether this can be attributed to increased lipofuscin or to increased calcium deposition [5]. FAF is not very useful in demonstrating *peau d’orange* [5]. This can be explained by the fact that *peau d’orange* stems from alterations at the level of the Bruch’s membrane beneath the RPE [5]. Blue or green excitation light is not absorbed well at that level, whereas near-infrared light is [5,15]. Therefore, NIR is more suitable for demonstrating *peau d’orange* compared to FAF [5,8,15,62]. Finally, the areas of chorioretinal atrophy exhibit marked hypo-autofluorescence and appear dark on FAF imaging [5] (Figure 2). This makes FAF a very useful tool for documenting chorioretinal atrophy and monitoring its extension with serial imaging [5]. 

**(III) OPTICAL COHERENCE TOMOGRAPHY (OCT)**.

**Figure 3:**
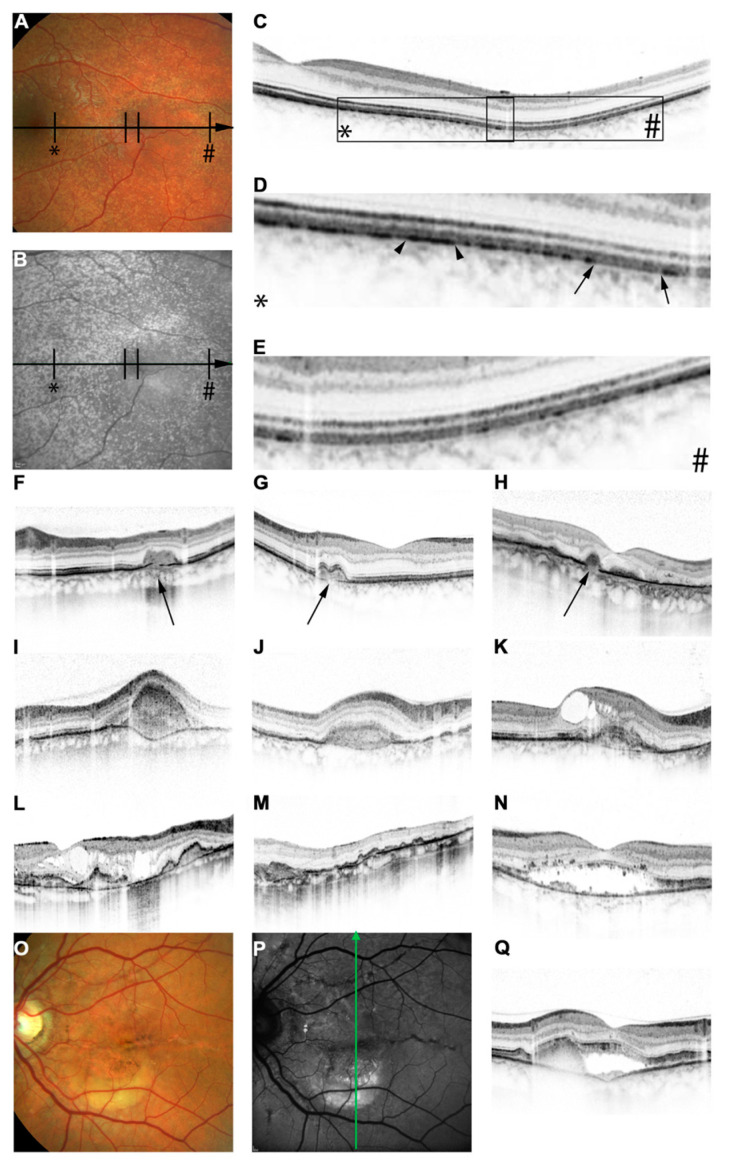
Features of pseudoxanthoma elasticum on optical coherence tomography (OCT). Calcification of Bruch’s membrane may be seen on OCT images. This is best illustrated within areas of peau d’orange, the transition zone between the calcified and un-calcified Bruch membranes (**A**–**E**). The horizontal arrow in (**A**,**B**) indicates the placement of the OCT scan in (**C**). Magnifications (2.5×) of characteristic details in (**C**) are shown in (**D**,**E**). Area * corresponds to (**D**) and area # to (**E**). Areas of increased reflectivity within the outer zone of the RPE–Bruch’s membrane complex (arrow heads in (**D**)) correlate with the whitish opaque fundus reflex on color images (**A**) and the increased signal on near-infrared reflectance images (**B**). Areas of lower reflectivity ((**E**), arrows in (**D**)) correlate to the normal fundus reflex. Angioid streaks correlate to breaks within the thickened and hyperreflective Bruch’s membrane ((**F**–**H**), arrows). Fibrovascular tissue may grow through such breaks (**I**,**J**). A typical complication of angioid streaks is the development of choroidal neovascularization leading to retinal exsudation (**K**). Eventually, atrophy of the retinal pigment epithelium is associated with atrophic changes in the photoreceptor layer with (**L**) or without (**M**) cystoid retinal lesions. In some patients there may be persistent subretinal fluid independent of choroidal neovascularization (**N**,**Q**). If longstanding, a vitelliform lesion may present with deposition of yellowish hyperautofluorescent material at the bottom of the lesion (**O**–**Q**). The green arrow in (**P**) indicates the placement of the OCT scan in (**Q**) [5].

Spectral-domain OCT (SD-OCT) is also a non-invasive tool that can depict changes secondary to AS, including secondary CNV formation. Bruch’s membrane undulations (inward and outward deformations) [5] can be detected using an OCT scan [5] (Figure 3). They seem to be more prominent in PXE eyes than AMD patients [5,72], and this may be an important biomarker to distinguish between CNV due to streaks and conventional wet AMD-related CNV [5,72]. They are usually located around the optic nerve and are thought to be caused by stretching forces around the optic nerve [3,73]. It has been suggested that undulations precede breaks at the level of the Bruch’s membrane and may in fact serve as precursors of such breaks where CNVs develop [3].

Focal calcifications of the Bruch’s membrane appear as areas of hyper-reflectivity on the OCT scan [3,15,74,75]. Pattern dystrophy-type changes may also appear as the deposition of hyper-reflective material, which can be seen either at the RPE level or below or above it [5,15,76].

OCT is very good at detecting sub-retinal or intra-retinal fluid associated with CNV formation [5]. In addition, it is a very good tool to monitor the response to anti-VEGF therapy by observing a reduction or complete resolution of fluid [5]. However, in late stages, atrophic lesions can be accompanied by the presence of cystic spaces, which is very difficult to distinguish from active fluid [5]. Another interesting point is that OCT may be able to detect sub-retinal fluid in the absence of an active CNV in some PXE patients [5,76]. This OCT finding may resemble central serous retinopathy (CSR) [5,76,77] (Figure 3). It is proposed that the fluid in these cases may have appeared due to RPE pump dysfunction, increased hydrophobicity of the Bruch’s membrane, or both [5,77]. Limited data suggest that this type of sub-retinal fluid does not respond to anti-VEGF agents or carbonic anhydrase inhibitors [5,77]. In chronic cases, the persistence of this non-CNV-associated sub-retinal fluid may result in the development of a vitelliform lesion that will appear markedly hyper-reflective on an OCT scan and will demonstrate increased hyper-autofluorescence on FAF imaging [5]. 

Comet lesions are usually located in the peripheral retina, whereas OCT is mainly focused on depicting the macular region. Therefore, OCT is not widely used to detect comet lesions [5]. From the limited available data, comet lesions appear to be hypo-reflective cystic spaces that involve the outer retinal layers with a hyper-reflective inner lining and focal debris-like deposits just above the RPE level [5,15] (Figure 3). 

The OCT can also provide information about the choroid [3,5,72]. Ellaban et al. reported that eyes with ASs and no CNV had similar choroidal thickness as the normal controls [3,72]. However, in the same manuscript, Ellaban et al. reported that patients with ASs with CNV formation had thinner choroids than the normal controls [3,5,72]. 

**(IV) OCT ANGIOGRAPHY (OCT-A).**

OCT-A is a non-invasive imaging modality that is used to assess the retinal and optic disc vasculatures. It allows for the detection of the presence of CNV in a wide range of retinal conditions. Furthermore, it evaluates the blood perfusion of the macula and the optic nerve head. This technology emerged from the OCT and was granted Food and Drug Administration (FDA) approval for wider use in ophthalmic practice in 2015 [78]. Multiple sequential B-scans are carried out repeatedly over a specific part of the retina at high speed [78]. This allows the distinction between areas with high blood flow and areas with slow or no flow and gives information about the location of the abnormal blood flow [78].

OCT-A has gained increased popularity over the past decade compared to the conventional angiographic imaging modalities, including fundus fluorescein angiography (FFA) and indocyanine green angiography (ICG-A). Unlike FFA and ICG-A, it does not require the intravenous injection of a dye, and this eliminates the possibility of potential adverse events linked with the use of a dye (for example, nausea, vomiting, rash, or anaphylactic reaction) [78]. Therefore, it is much safer and better tolerated by patients compared to conventional retinal angiography imaging techniques [78]. It is also less time-consuming compared to FFA and ICG-A [78]. In addition, it seems that the lack of a dye produces better-quality, enhanced-depth imaging of the retinal and choroidal vasculature [78,79,80,81,82,83]. All these advantages have allowed the wider use of OCT-A in clinical practice for a broad range of retinal conditions, including the study of ASs and the formation of secondary CNV.

**Figure 4:**
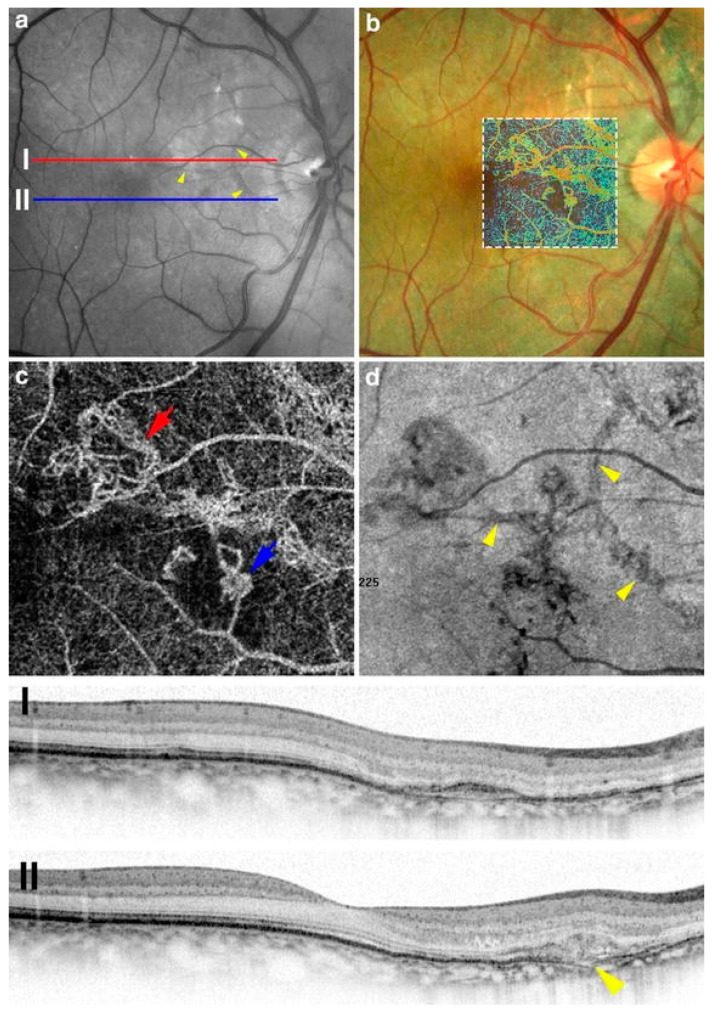
Multimodal imaging findings of choroidal neovascularization (CNV). Angioid streaks are observed on the red-free image (**a**); however, no hemorrhage is evident. The overlay of false-colored optical coherence tomography angiography (OCT-A) over the color photograph (**b**) outlines the area of choroidal neovascularization (CNV). En Face OCT-A (**c**) demonstrates two areas of CNV (red and blue arrows) that demonstrate a tangled morphology of vascular networks. Sites of CNV closely correlate to sites of angioid streaks ((**d**); arrowheads) when OCT-A is compared to face reflectance images. Structural OCT scans (**I**,**II**) confirm a mixed type 1 (**I**) and type 2 (**II**) neovascular lesion that arises in proximity to sites of Bruch’s membrane disruption [47].

Gal-Or et al. were the first to describe OCT-A findings in a PXE patient with ASs and secondary CNV formation [47]. The authors reported that the features of the CNV followed the path of the streak [47]. These findings from a novel imaging technique at that time highlighted the implication of Bruch’s membrane in the formation of ASs even further [3,47] (Figure 4). Other reports during the following years described a deep irregular vascular network that may represent fibrovascular tissue over the dehiscent areas of the Bruch’s membrane [3,84,85,86,87]. The existence of an irregular vascular network is proposed to be a biomarker of CNV activity [86,87]. In their case series of 19 AS patients with secondary CNV (38 eyes), Corbelli et al. reported that all CNVs exhibiting a tangled appearance on OCT-A showed no signs of activity on the additional multimodal imaging [85]. On the contrary, in the same case series, the majority of CNVs with interlacing appearances seemed to show activity [85]. On the other hand, Falfoul et al. reported limitations in accurately assessing CNV activity in their case series of 16 patients (31 eyes) [86]. These reports are predominantly retrospective in nature, with a small number of eyes and limited follow-up. As a result, more prospective studies with longer follow-ups and a larger number of studied patients will be required to validate some of the observations in these reports and to construct a more robust and reliable algorithm for the interpretation of OCT-A in such patients. In addition, none of these reports relied exclusively on OCT-A [85,86]. Finally, Marchese et al. commented that OCT-A might have very high sensitivity and specificity in detecting secondary CNV, but OCT-A by itself cannot reliably predict CNV activity [88]. It appears that OCT is the best predictor of CNV activity [88]. The presence of sub-retinal hyper-reflective material (SHRM) seems to correlate well with CNV activity in a similar fashion as with CNV due to wet AMD [88,89,90,91,92,93]. This suggests that the OCT-A findings should be carefully correlated with the findings of other imaging modalities, namely OCT, FFA, and ICG-A [85,86,88].

Moreover, OCT-A has enabled the observation of structural changes in the choriocapillaris in patients with ASs. Corbelli et al. and Falfoul et al. independently reported reduced density of the choriocapillaris vascular network [85,86]. This was further supported by a study published last year by Le et al. [94]. Nevertheless, Le et al. acknowledge that their study has significant limitations, including its cross-sectional and retrospective nature and the limited number of eyes included [94]. In addition, they commented on the fact that the eyes included in the study were treated with anti-VEGF agents, which might interfere with the results [94]. Le et al. suggested that longer longitudinal prospective data are needed to further validate their own observations [94].

In summary, OCT-A is useful in the detection of CNV, but it is not reliable on its own to confirm CNV activity.

Therefore, clinicians should not depend solely on OCT-A to make clinical decisions regarding the treatment of patients with CNV secondary to ASs, but they should combine the OCT-A findings with other imaging techniques.

**(V) FUNDUS FLUORESCEIN ANGIOGRAPHY (FFA).**

FFA is based on the principle of fluorescence, which is the ability of a substance to emit light of a longer wavelength when excited by light of a shorter wavelength [1]. Fluorescein, when stimulated by blue light, produces a light in the yellow-green spectrum [1]. Both blue and yellow–green wavelengths are within the visible spectrum of light [1]. The light emitted by the excited fluorescein molecules is captured by a film in the FFA apparatus, and the signal is then reconstructed to give an assessment of the retinal vasculature [1]. FFA plays a pivotal role in the confirmation of the existence of a secondary CNV [3,95,96,97,98]. Nevertheless, in asymptomatic AS patients, it does not add much more to the diagnosis or treatment [5].

**Figure 5:**
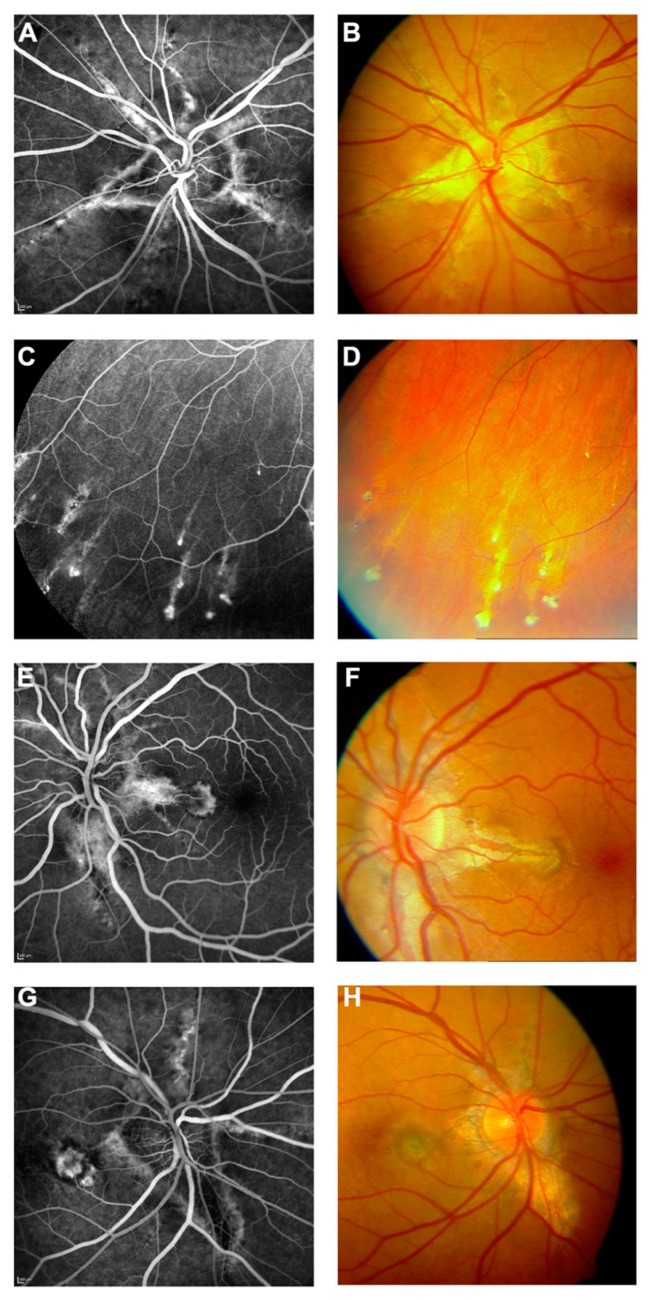
Features of pseudoxanthoma elasticum on fluorescein angiography. Angioid streaks typically show variable staining on fluorescein angiography (**A**,**B**). Comet tail lesions appear as hyperfluorescent spots with their tail toward the optic disk (**C**,**D**). Choroidal neovascularizations are mostly classic membranes. Sometimes, their detection may be difficult due to adjacent staining of angioid streaks (**E**–**H**) [5].

Since ASs represent breaks at the Bruch’s membrane, this allows more visibility of the underlying choroidal circulation, and this appears as an area of hyper-fluorescence at the early FFA stages that is known as a window defect [1,3]. In addition, when an active secondary CNV is present, FFA will demonstrate leakage [1]. Both classic and occult CNVs can occur with ASs. Classic CNV is located between the retina and the RPE and will appear as an area of lacy hyper-fluorescence, whereas occult CNV is located beneath the RPE and shows stippled hyper-fluorescence [1]. Finally, when a scar is present, there will be hyper-fluorescence evident at the last stages, which is known as late staining [1]. Wide-field FFA may detect comet lesions that usually appear to be hyper-fluorescent at the late stages (Figure 5). Retinal hemorrhages will be hypofluorescent due to the masking effect of blood [1].

However, it must be pointed out that, in longstanding cases of CNV, late staining might be confused with actual leakage, and therefore interpretation of FFA imaging might be challenging [88]. Furthermore, FFA is more time-consuming, and there are some side effects associated with the intravenous injection of fluorescein, including nausea, vomiting, skin discoloration, an anaphylaxis [78]. Despite the side effects of fluorescein and its limitations in imaging the retina, FFA still remains the gold standard of investigation for the detection of CNV [5].

**(VI) INDOCYANINE GREEN ANGIOGRAPHY (ICG-A).**

ICG-A is superior to the FFA in terms of delineating the choroidal vascular network, as it can circumvent the masking effect of the RPE [1]. Unlike FFA, near-infrared light is used (excitation wavelength: 805 nm, emission wavelength: 835 nm) [1]. In addition, indocyanine green has a much higher binding affinity with albumin and the rest of serum proteins, which can reach up to 98%, whereas the binding affinity of fluorescein reaches up to 80% [1]. In addition, the size of indocyanine molecules is greater than the size of fluorescein molecules [1]. As a result, indocyanine green dye stays within the choroidal vascular network, which allows for better depiction and imaging of the choriocapillaris [1]. Finally, near-infrared light is scattered less than visible light [1]. This property of infrared light mitigates the effect of any media opacities and allows for clearer imaging [1]. All the above properties make ICG-A more suitable and superior to FFA to delineate ASs in most cases [5,99].

ICG-A shows four different patterns in AS: (i) fluorescent; (ii) hypofluorescent; (iii) “track-like”; and (iv) mixed [3,99]. According to Lafaut et al., the first out of the four patterns is the most common [3,99]. However, there is a significant percentage of ASs reaching up to 20% that is visible on dilated fundus examination but may not be demonstrated on ICG-A [3]. Furthermore, occult CNVs are better visualized with ICG-A than with FFA [3].

**Figure 6:**
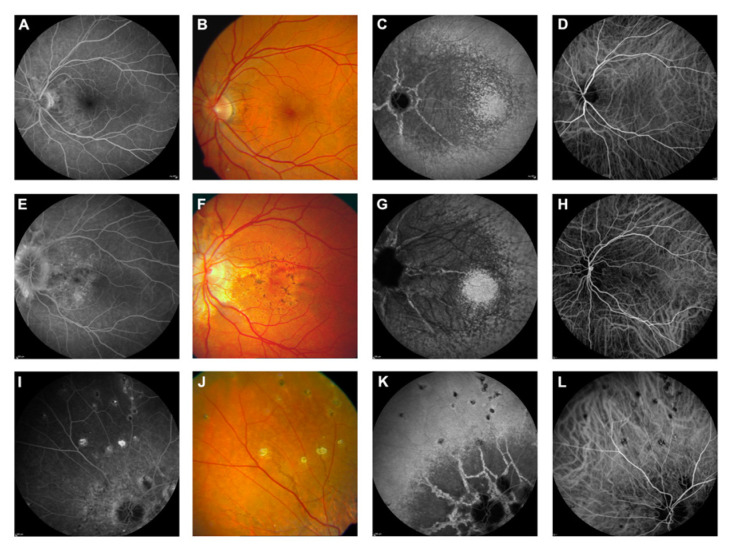
Comparison of clinical features on late phase fluorescein angiography, early and late phase indocyanine green angiography (ICG-A), and funduscopy. Late-phase fluorescein angiography shows variable staining of angioid streaks (**A**,**E**,**I**), which corresponds well with findings on funduscopy (**B**,**F**,**J**). A characteristic finding on late-phase ICG-A is a centrally reduced fluorescence with a spotted transition zone to normal peripheral fluorescence (**C**,**G**,**K**). Angioid streaks are well visible within the dark, non-fluorescent area. Note that there is no correlation between color images (**B**,**F**,**J**) or early ICG-A frames (**D**,**H**,**L**). Comet tail lesions (**J**) are usually hyperfluorescent on late-phase fluorescein angiograms (**I**) and hypofluorescent on ICG late-phase angiograms (**K**) [5].

In PXE, ASs are better visualized towards the late phases of ICG-A and appear hyperfluorescent [5] (Figure 6). Another typical feature in an ICG-A of a PXE patient is that the posterior pole usually demonstrates reduced late phase fluorescence [5,52]. On the contrary, the more eccentric areas demonstrate normal late fluorescence [5]. This leaves a transitional zone between these two regions, which is more prominent at the temporal retina most of the time [5] (Figure 6). According to Charbel Issa [8] and Charbel Issa et al. [52], this is an imaging phenomenon observed at the late ICG-A phases that does not match with the areas of *peau d’orange* and therefore does not represent *peau d’orange* [8,52]. Finally, comet lesions exhibit hypofluorescence during the late phases of ICG-A [5] (Figure 6). Gliem et al. propose that, because of the invasive nature of ICG-A, it should be reserved for monitoring for polyps or occult CNV or for studying the pathophysiological mechanisms of the development of ASs in PXE [5]. They also recommend that it not be used routinely for the monitoring of AS patients [5].

### 3.8. Treatment of ASs

To date, there is no definitive treatment for ASs [3]. The focus of current therapies is to treat secondary CNV and keep it inactive so that preservation of vision can be achieved [3]. The various treatments for the treatment of CNV will be discussed in greater detail below.

Before the adoption of anti-VEGF therapies, argon laser photocoagulation was utilized as a treatment for angioid streaks (ASs), specifically for juxta-foveal and extra-foveal lesions. However, its use was controversial due to mixed outcomes and a tendency to cause retinal tissue damage, sub-retinal hemorrhages, and vision reduction, leading to its discontinuation [3,100,101,102,103,104,105,106]. Similarly, ICG-guided photothrombosis for sub-foveal CNV showed some promise with stable acuity after 12 months in a limited case series, but due to the risks of foveal damage and a lack of broader evidence, it has not been widely accepted [3,107].

Transpupillary thermotherapy and invasive surgical approaches, including macular translocation and subretinal extraction, also failed to offer significant benefits because of the associated frequent CNV recurrences, rendering these methods obsolete in current treatment strategies for ASs [3,108,109,110,111,112,113,114,115]. Photodynamic therapy (PDT), initially adopted based on early successes in treating classic CNV from wet AMD, was later explored for AS-related CNV. Despite initial optimism, subsequent studies indicated that PDT necessitated multiple sessions and could exacerbate RPE and Bruch’s membrane damage, leading to disease recurrence and poor outcomes, thus falling out of favor for this application [3,116,117,118,119,120,121,122,123,124,125,126,127,128,129,130,131,132].

The introduction of anti-VEGF therapy significantly altered the visual outcome in patients who develop CNV secondary to wet AMD and due to other causes [3,133,134]. Inevitably, anti-VEGF agents were used for the treatment of ASs and secondary CNV as well.

The three main anti-VEGF agents that have been used as monotherapy for the treatment of CNV secondary to ASs are ranibizumab, aflibercept, and off-label bevacizumab [135,136,137,138,139,140,141,142,143,144,145,146,147,148,149,150,151,152,153,154,155,156,157,158,159,160,161,162,163,164,165,166,167,168,169,170]. Numerous studies and reports have been published describing the visual and anatomical outcomes of these three anti-VEGF agents [135,136,137,138,139,140,141,142,143,144,145,146,147,148,149,150,151,152,153,154,155,156,157,158,159,160,161,162,163,164,165,166,167,168,169,170]. None of these reports were randomized, double-blind trials. They were either retrospective case reports or case series with a variable number of eyes enrolled and variable follow-up [135,136,137,138,139,140,141,142,143,144,145,146,147,148,149,150,151,152,153,154,155,156,157,158,159,160,161,162,163,164,165,166,167,168,169,170]. Nevertheless, they all demonstrate that all three anti-VEGF agents manage to at least stabilize the visual acuity regardless of the underlying systemic cause of ASs [3,135,136,137,138,139,140,141,142,143,144,145,146,147,148,149,150,151,152,153,154,155,156,157,158,159,160,161,162,163,164,165,166,167,168,169,170]. They also achieve a reduction in retinal thickness, but this does not necessarily translate into an improvement in visual outcome [3,139]. Administration of anti-VEGF did not seem to result in the conversion of the treated CNV into a scar [3,139]. In addition, in eyes previously treated with ranibizumab that failed to respond to treatment, switching to aflibercept seems to be a safe and viable option [155]. Finally, treatment of juxta-foveal and extra-foveal CNVs seems to demonstrate better visual outcomes compared to sub-foveal CNVs [147].

The authors of this review article also found one single case report of a case with CNV secondary to ASs that was treated successfully with one single dose of intravitreal brolucizumab [171]. However, as the authors of this article acknowledge, this is a retrospective single-case report with limited follow-up [171]. Therefore, larger prospective studies will be required to elucidate the safety and efficacy of brolucizumab in the treatment of CNV secondary to ASs [171].

Recently, the use of a novel anti-VEGF agent called faricimab has been introduced into clinical practice, especially for the treatment of wet AMD and diabetic macular oedema [172,173]. The real-life results of faricimab seem to be promising [174,175,176]. Due to the very recent introduction of this novel therapeutic agent and to the best of our knowledge and literature search, there are no case reports or case series (retrospective or prospective) describing real-life experience with the use of faricimab against CNV secondary to ASs. As faricimab seems to be gaining increasing popularity, it is most likely only a matter of time before such reports are published. It would be quite interesting to see the short-term and long-term results of the application of this novel anti-VEGF drug against this rare cause of secondary CNV.

Regarding the safety profile of the administration of anti-VEGF agents, the available data so far show no major concerns [3,139]. A pertinent topic of concern is the theoretical risk of cerebrovascular and cardiac events in patients undergoing anti-VEGF therapy, especially those who have been diagnosed with PXE [3]. However, from the available data, there does not seem to be an increased frequency of such events in AS patients [3,177].

Another interesting point is whether a pro re nata (PRN) or treat and extend (T&E) should be adopted to maximize the efficacy of the administered anti-VEGF therapeutic agent. There is no unanimous treatment algorithm. Other groups recommend an initial loading with injections at 4-weekly intervals, followed by a PRN injection pattern [3,139]. Other treatment groups have suggested that a fixed-dose regimen might be better suited in cases where close follow-up is not possible [3,54]. Our literature search yielded only one case report of a patient with ASs that was treated with a T&E protocol [152]. Ideally, larger prospective, randomized, and double-blind control clinical trials should be conducted to elucidate which treatment pattern is superior for this rare type of secondary CNV and which of the available anti-VEGF agents should be better. Nevertheless, such trials are very difficult to design and come into fruition due to the paucity of AS patients, which will limit the power of such endeavors and their cost-effectiveness.

Combinations of different treatment modalities have also been reported. A combination of PDT with bevacizumab [3,178,179] has been described, but the results were no different from anti-VEGF monotherapy overall [3,178,179]. Finally, there is a single case report of the combination of PDT with intravitreal triamcinolone [3,180], which did not yield a more favorable result in comparison to PDT monotherapy [3,180].

## 4. Future Directions

The evolving understanding of ASs underscores the necessity for ongoing research, particularly in the realms of genetic predisposition and molecular mechanisms. Advances in genetic engineering and gene therapy offer promising avenues for comprehending the pathogenesis of ASs and developing targeted treatments. Future research could focus on elucidating the specific genetic mutations and their functional impacts in conditions like PXE and related disorders. Additionally, exploring the molecular pathways involved in the mineralization of Bruch’s membrane could yield novel therapeutic targets. The potential of utilizing advanced imaging techniques, such as OCT-A, for early detection and monitoring of disease progression also warrants further investigation.

Another crucial area for future research is the development and refinement of treatment strategies for complications associated with ASs, such as CNV. While current treatments primarily focus on anti-VEGF therapies, there is scope for exploring new pharmacological agents and combination therapies. Research into personalized medicine approaches, tailored to individual genetic profiles and disease phenotypes, could significantly enhance treatment efficacy. Furthermore, long-term studies are needed to better understand the natural history of ASs and to develop guidelines for monitoring and management, especially in patients with systemic associations. Collaborative efforts, including multi-center studies and international registries, would be instrumental in advancing our knowledge and improving patient outcomes in this complex field.

## 5. Conclusions

ASs are characterized by brittle lesions in Bruch’s membrane, often linked to systemic conditions with genetic components like PXE [3,8]. A multidisciplinary approach is essential for comprehensive patient care. Various imaging techniques, each with unique benefits and limitations, are crucial in treatment decision making. CNV presents the most significant risk to vision in AS patients, with anti-VEGF treatments offering variable outcomes [3,5]. Patients should be informed about CNV risks, advised on regular self-monitoring, and cautioned against activities risking ocular trauma [5,181]. The advent of anti-VEGF therapies, particularly faricimab, has notably improved visual outcomes in CNV, with future studies expected to further confirm its benefits. Additionally, insights into the ABCC6 gene could pave the way for novel preventative therapies targeting Bruch’s membrane calcification.

## Data Availability

The data presented in this study are available in this article.

## Abbreviations

Angioid streaks (ASs); central serous retinopathy (CSR); choroidal neovascular membrane (CNV); Ehlers–Danlos (ED); fundus autofluorescence (FAF); fundus fluorescein angiography (FFA); indocyanine green angiography (ICG-A); photodynamic therapy (PDT); pro re nata (PRN); pseudoxanthoma elasticum (PXE); retinal pigment epithelium (RPE); sub-retinal hyper-reflective material (SHRM); treat and extend (T&E); vascular endothelial growth factor (VEGF).
